# Genomic Analysis of Three Cheese-Borne *Pseudomonas lactis* with Biofilm and Spoilage-Associated Behavior

**DOI:** 10.3390/microorganisms8081208

**Published:** 2020-08-08

**Authors:** Laura Quintieri, Leonardo Caputo, Maria De Angelis, Francesca Fanelli

**Affiliations:** 1 Institute of Sciences of Food Production, National Research Council of Italy, Via G. Amendola 122/O, 70126 Bari, Italy; laura.quintieri@ispa.cnr.it (L.Q.); francesca.fanelli@ispa.cnr.it (F.F.); 2 Department of Soil, Plant and Food Science, University of Bari Aldo Moro, Via Amendola 165/a, 70126 Bari, Italy; maria.deangelis@uniba.it

**Keywords:** biofilm, genomics, mozzarella cheese, phylogenetic analysis, pigments, proteolysis, lipolysis

## Abstract

Psychrotrophic pseudomonads cause spoilage of cold fresh cheeses and their shelf-life reduction. Three cheese-borne *Pseudomonas* sp., ITEM 17295, ITEM 17298, and ITEM 17299 strains, previously isolated from mozzarella cheese, revealed distinctive spoilage traits based on molecular determinants requiring further investigations. Genomic indexes (ANI, *is*DDH), MLST-based phylogeny of four housekeeping genes (16S rRNA, *gyr*B, *rpo*B and *rpo*D) and genome-based phylogeny reclassified them as *Pseudomonas lactis*. Each strain showed distinctive phenotypic traits at 15 and 30 °C: ITEM 17298 was the highest biofilm producer at both temperatures, whilst ITEM 17295 and ITEM 17299 showed the strongest proteolytic activity at 30 °C. A wider pattern of pigments was found for ITEM 17298, while ITEM 17295 colonies were not pigmented. Although the high genomic similarity, some relevant molecular differences supported this phenotypic diversity: ITEM 17295, producing low biofilm amount, missed the *pel* operon involved in EPS synthesis and the biofilm-related Toxin-Antitoxin systems (*mqs*R*/mqs*A, *chp*B*/chp*S); *pvd*S, required for the pyoverdine synthesis, was a truncated gene in ITEM 17295, harboring, instead, a second *apr*A involved in milk proteolysis. This work provided new insight into the food spoiler microbiota by identifying these mozzarella cheese spoilers as *P. lactis*; molecular targets to be exploited in the development of novel preservative strategies were also revealed.

## 1. Introduction

The genus *Pseudomonas* comprises species naturally widespread in the environment, including human pathogens, such as the clinically relevant *P. aeruginosa* [1,2]. *Pseudomonas* species displayed a large array of ecological functions, i.e., plant-growing promoter, bioremediation, biodegradation, and biosorption, conferring them high ecological and biotechnological importance [3,4,5]. Its taxonomy was lastly updated in 2018 by Peix et al. [6] who listed further 70 species described in the last few years in addition to the 128 previously described the same authors in 2009 [7]. However, genus taxonomy is currently discussed; indeed, according to the List of Prokaryotic Names with Standing in Nomenclature (https://lpsn.dsmz.de/genus/pseudomonas), the genus *Pseudomonas* (family *Pseudomonadaceae*, class *Gammaproteobacteria*) comprises 278 child taxa with a validly published name, including synonyms, among which 114 were represented by at least one type strain (WFCC Global Catalogue of Microorganisms; http://gcm.wfcc.info/overview/).

The re-definition of *Pseudomonas* taxonomy has recently been made possible by the availability of a plethora genomic data and the delineation of standards to describe new bacterial species [8,9]. Phylogenetic studies performed by using genomic indexes (ANI, DDH) and/or multilocus sequence typing (MLST) of housekeeping genes (16S rRNA gene, *gyr*B, *rpo*B, *rpo*D), which were proved to have a high discriminatory power in defining *Pseudomonas* taxonomy [10,11], have led to the reconstruction of pseudomonads into two main separate lineages: the *P. aeruginosa* lineage comprising three main groups (*P. aeruginosa*, *P. stutzeri*, and *P. oleovarians*), and the *P. fluorescens* lineage including six groups (*P. fluorescens*, *P. syringae*, *P. lutea*, *P. putida*, *P. angiulliseptica*, and *P. straminea*).

Recently, von Neubeck et al. [12] described two novel *Pseudomonas* spp., *P. lactis*, and *P. paralactis*, isolated from bovine raw milk, which differentiated from their closest genetic-relatives for the phenotypic and chemotaxonomic properties, such as the assimilation or production of acid from carbohydrates. Both *P. lactis* and *P. paralactis* were included in the *P. fluorescens* subgroup by MLST-based phylogeny [12,13]. Likewise, for *P. fluorescens* strains, they showed proteolysis of skimmed milk at 25 and 4 °C, lipolytic activity towards tributyrin at 4 °C and fluorescent pigmentation on King B; metabolic and growth characteristics were also reported [12].

Since they are well adapted to the low temperatures, psychrotrophic *Pseudomonas* species are associated with spoilage of fresh dairy products during cold storage [14,15,16]; food spoilage is caused by proteolytic and lipolytic activities, off-flavors (due to volatile compounds production and amino acid catabolism), and pigment release [15,16,17,18,19,20]. It has been reported that biofilm plays a role in spoilage by promoting enzyme production and color development [21,22]. Furthermore, biofilm eradication from the dairy environments is generally difficult since they are resistant to common antimicrobial strategies [23,24,25] and, thus, they provided a persistent source of contamination.

The world’s most popular Italian Mozzarella cheese is a high-moisture dairy product showing a short shelf life due to the high load of autochthonous psychrotrophic *Pseudomonas* spp. [14,17,26,27]; although widely investigated, the preservation strategies developed so far and addressed to counteract these bacteria on mozzarella cheese and similar products have been scarcely transferred [16]. Thus, a deep investigation of its spoilage microbiota can lead to the development of new screening methodologies and procedures useful to counteract the spread of spoilage pseudomonads [22,28,29,30]. In light of these considerations, in this work, three food-borne *Pseudomonas* spp. spoilers, previously isolated from spoiled mozzarella cheese [14,17], were genomically characterized aiming at improving their taxonomic placement; then, phenotypic traits underlying the adaptation, persistence and spoilage were also revealed.

## 2. Materials and Methods

### 2.1. Strains and Culture Condition

*Pseudomonas* spp. strains ITEM 17295, ITEM 17298 and ITEM 17299 were originally isolated from high-moisture mozzarella cheese [14,15] and maintained at −80 °C as pure stock cultures in Nutrient Broth (NB; Oxoid S.p.A., Rodano, Milan, Italy) supplemented with glycerol 30% (*v*/*v*). The strains were routinely refreshed (30 °C, 24 h) by streaking onto Luria Bertani (LB; Sigma Aldrich, Milan, Italy) agar, and cultivated into 5 mL of LB broth for 16 h at 30 °C and 150 strokes to prepare the bacterial inocula for the subsequent experiments.

### 2.2. Genome Sequencing and Assembly

The DNA was extracted from a single colony of *Pseudomonas* spp. strains by using the Wizard^®^ Genomic DNA Purification Kit (Promega). The integrity, purity and quantity of DNA were assessed as previously described by Fanelli et al. [31] and submitted to IGA Technology Services (Udine, Italy) for whole-genome shotgun sequencing using the Illumina HiSeq2500 platform.

A preliminary evaluation of the quality of the raw data was performed with FastQC software. De novo assemblies were performed using SPAdes version 3.5.0 software within the Galaxy platform [32]. The overall contiguity of the assembly and genomes statistics were determined with MIGA [33].

### 2.3. Bioinformatic Methods

Genes were predicted and annotated using the PROKKA pipeline implemented in the Galaxy platform (Galaxy Tool Version 1.0.0 [32]). The predicted proteins were submitted to the PFAM annotator tool within the Galaxy platform in order to predict the Pfam domains. Protein ID used in the manuscript indicated those obtained by NCBI (National Center for Biotechnology Information) Prokaryotic Genome Annotation Pipeline [34].

Predicted proteins were assigned to Clusters of Orthologous Groups (COG) functional categories by Web CD-Search Tool [35] using an Expected value threshold of 0.01. Then, COG ID were then manually mapped into functional categories (https://www.ncbi.nlm.nih.gov/COG/).

All the protein sequences used in this study were retrieved from GenBank (NCBI). The homology-based relationship of ITEM 17295, ITEM 17298, and ITEM 17299 predicted proteins towards selected proteins was determined by the BLASTP algorithm on the NCBI site (http://blast.ncbi.nlm.nih.gov/Blast.cgi). Gene models were manually determined, and clustering and orientation were subsequently deduced for the closely linked genes.

Comparison of orthologous gene clusters among strains was performed by OrthoVenn2 [36]. Functional annotation, subsystem prediction, and metabolic reconstruction comparison were also performed using the RAST server [37] and by using The Proteome Comparison Service integrated in Patric (www.patric.org; [38]).

### 2.4. Phylogenetic Analysis

Sequences of the four housekeeping genes (16S rRNA, *gyr*B, *rpo*B and *rpo*D) were extracted from each genome by BLASTn search and compared to type strains sequences retrieved from Gomila et al. [10] and Mulet et al. [11]. The alignment of the concatenated dataset was performed using MUSCLE implemented in MEGA version 10.1.7 [39]. Phylogenies was inferred by using the Maximum-Likelihood (ML) method and Tamura-Nei model [40]. The tree was graphically generated by iTOL version 5.5 [41].

Genetic divergence was calculated by the ANI/AAI calculator [42,43] which estimates the average nucleotide/amino acid identity (ANI/AAI) using both best hits (one-way ANI) and reciprocal best hits (two-way ANI) between genomic datasets. The Genome-to-Genome Distance Calculator (GGDC) [44,45] web service was used to report digital DDH for the accurate delineation of prokaryotic subspecies and to calculate differences in G+C genomic content (available at ggdc.dsmz.de). Formula 2 alone was used for analysis, providing an estimation of DDH independent of genome lengths, as recommended by the authors of GGDC for use with any incomplete genomes [44,46].

Genome-based phylogeny was constructed by using The Phylogenetic Tree Building Service implemented in Patric platform (www.patric.org) considering the *Cellvibrio japonicus* Ueda 107 as outgroup according to Gomila et al. [10] and Maximum Likelihood method RAXML with progressive refinement [47]. For each strain, genomic sequences deposited in GenBank were used for the analysis.

### 2.5. Phenotypic Analysis under Two Temperatures of Incubation

Due to the high versatility and adaptation mechanisms of *Pseudomonas* spp., phenotypic traits were investigated both at the optimum growth temperature (30 °C) and at lower temperature (15 °C) in order to mime cold storage conditions that could favor their persistence and spoilage traits in dairy industry.

#### 2.5.1. Static Biofilm Formation and Motility Assays

Biofilm formation was assayed in 96-well microtiter plates and quantified as described by O’Toole [48]. Briefly, overnight cultures of *Pseudomonas* spp. ITEM 17295, ITEM 17298, and ITEM 17299 were diluted 1:100 into fresh LB (100 µL; 8 replicates for each time point) and incubated at 15 and 30 °C for 192 h. At 24, 48 h, 72 h, and 192 h, planktonic cells were carefully removed, and wells were washed twice with distilled water; biofilm cells adhering to the bottom and side of each well were stained with crystal violet (CV; 0.1%, *w*/*v*). After a second washing step, biofilm-associated crystal violet (CV) was solubilized with 30% acetic acid (*v*/*v*) and its optical density was measured at 570 nm. Swarming and swimming motility were performed in Petri dishes (polystyrene, diameter of 50 mm) containing 10 mL of LB solidified with 0.5 and 0.3% (*w*/*v*) of agar, respectively. Swim and swarm plates were inoculated with 2.5 µL of bacterial broth culture representing approximately 1 × 10^8^ CFU/mL (corresponding to 0.3 OD_λ = 600 nm_; [17]) as reported by Quintieri et al. [22]. All plates were incubated at 15 °C and 30 °C. The diameters of the swarming and swimming motility zones were measured at 24, 48, and 72 h of incubation. By contrast, twitching motility was evaluated on M8 minimal medium [49] supplemented with 1 mM MgSO_4_, 0.2% glucose (*w*/*v*), 0.5% casamino acids (*w*/*v*) and 1% agar (*w*/*v*). Bacterial cells were inoculated at the bottom of the agar–dish interface. The plates were incubated at 15 °C and 30 °C. At selected times (24, 48 and 72 h), the agar layer was carefully removed and the plates were stained with 0.1% of CV (*w*/*v*). After the washing step, the biofilm was solubilized and quantified as described above.

#### 2.5.2. Exopolysaccharides (EPS) Production by Congo Red Binding Assay

*Pseudomonas* spp. cultures were spotted (5 µL) onto Petri dishes containing Congo red agar (10 g/L tryptone, 40 µg/mL Congo red (Sigma), 15 µg/mL Coomassie brilliant and 1.5% agar) and incubated at 15 °C for 72 h. The interaction of Congo red with EPS produced the appearance of red colonies (biofilm producers). EPS production was also detected in the swimming assay performed in LB supplemented with 40 µg/mL Congo Red and 15 µg/mL Coomassie brilliant, as described above.

#### 2.5.3. Proteolytic and Lipolytic Activity

Fresh cultures of *Pseudomonas* spp. ITEM 17295, 17298, and 17299, routinely grown on PCA medium (Biolife) for 16 h, were patched in triplicate by toothpick onto Petri dishes with Milk agar (90% mL UHT skimmed milk added to 10% ml of sterile 1.6% agar) and Rhodamine Agar [50] using olive oil (3.3% *v*/*v*) and 2 mL of filter-sterilized (0.22 µm) Rhodamine B solution (0.1% *w/v*) for screening proteolytic and lipolytic activity, respectively. The agar Petri dishes were incubated at 15 and 30 °C for 48 h. The diameters of clear or orange fluorescent halos (revealed under UV light at 360 nm) surrounding strain colonies on Milk Agar and Rhodamine Agar, respectively, were registered with a caliper.

#### 2.5.4. Pigment Production

Cell suspensions of each *Pseudomonas* spp. strain were spotted (2.5 µL; 1 × 10^8^ CFU/mL) in triplicate onto King A and King B agar Petri dishes (Sigma Aldrich) and Potato Dextrose Agar (PDA; [17]) and incubated at 15 and 30 °C for 1 week; pigmented colonies were also examined under Wood’s lamp.

### 2.6. Statistical Analyses

After assessing homogeneity variance by Levene’s test (*p* < 0.05), a three-or two-ANOVA was carried out using R software v. 3.6.3 with Rcmdr and RcmdrPlugin.aRnova packages to examine the effect time and temperature on biofilm biomass, swarming, swimming, and twitching produced by each assayed strain. Partial eta squared values (*η*^2^) were computed to analyze the effect size of the main factors and their interactions. Multiple comparisons among individual means were performed by HSD Tukey test (*p* < 0.05). When required, one-way ANOVA was performed followed by *post-hoc* HSD Tukey test (*p* < 0.05). In the case of unequal variances, the non-parametric Kruskal–Wallis H test was applied with Wilcoxon rank sum test and *p*-value adjustment Bonferroni’s method using the RcmdrPlugin.EZR package.

## 3. Results and Discussion

### 3.1. Genomic Analysis of Pseudomonas spp. Strains

#### 3.1.1. General Features of *Pseudomonas* sp. Genomes

*Pseudomonas* spp. strains genomes were sequenced using a whole genome shotgun approach. Genomes were assembled using Spades v5.0 software for a total of 89, 117, and 137 contigs (>500 bp) for ITEM 17295, ITEM 17298, and ITEM 17299, respectively. The average GC content is of 59.8% for all strain and the assembly length of 6.3 Mb (Table 1). The quality of the assemblies is excellent with 93.7% of genome completeness and N50 of 351 kb, 147 kb and 139 kb for ITEM 17295, 17298, and 17299, respectively). The whole genome shotgun projects have been deposited at DDBJ/ENA/GenBank under the accessions WJRV00000000 (for ITEM 17295), WJRU00000000 (for ITEM 17299) and NPKB00000000 (for ITEM 17298; [31]). The versions described in this paper are WJRV01000000 (for ITEM 17295), WJRU01000000 (for ITEM 17299), and NPKB01000000 (for ITEM 17298).

#### 3.1.2. Phylogenetic Analysis

The phylogeny of the isolates was initially determined by analyzing the 16S rRNA sequence retrieved from the genomic sequencing. 16S rRNA sequences of ITEM 17298 and ITEM 17299 are 100% similar to *P. paralactis* strain WS 4972 and 99.2% similar to *P. lactis* strain WS 4992; 16S rRNA sequence of ITEM 17295 shares 99.84% of identity to *P. paralactis* strain WS 4972 and 99.76% similar to *P. lactis* strain WS 4992.

**Figure 1:**
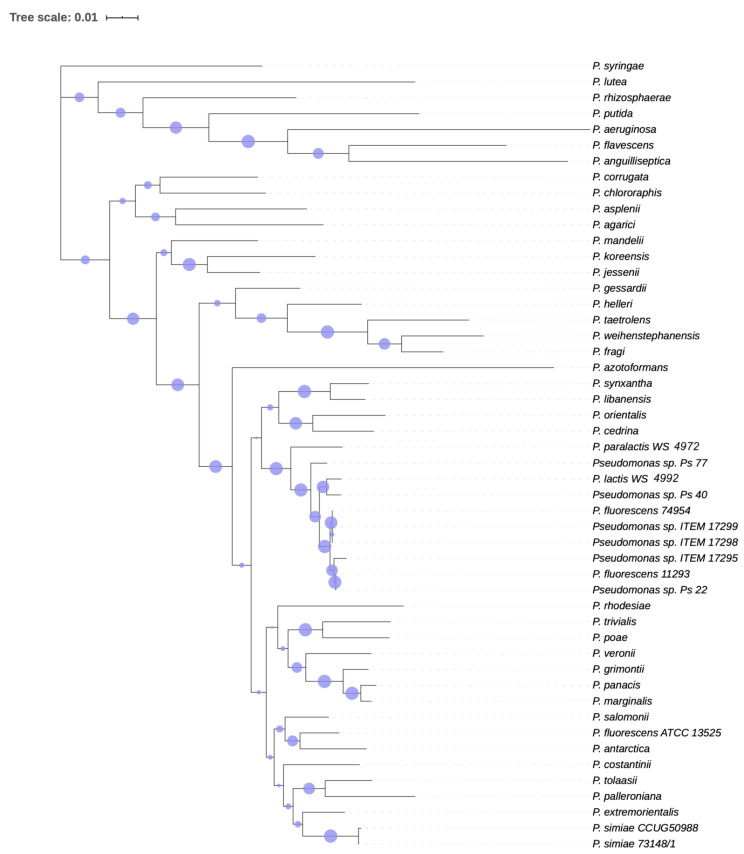
Phylogeny based on partial sequences of four housekeeping genes (16S rRNA, *gyr*B, *rpo*B and *rpo*D) was inferred by using the Maximum-Likelihood (ML) method and Tamura–Nei model [40]. Initial tree(s) for the heuristic search were obtained automatically by applying the neighbor-joining and BioNJ algorithms to a matrix of pairwise distances estimated using the Maximum Composite Likelihood approach, and then selecting the topology with a superior log likelihood value. The tree is drawn to scale, with branch lengths measured in the number of substitutions per site. Circles are representative of bootstrap values.

Since 16S rRNA is not adequate for discriminating *Pseudomonas* species as also previously reported [11], we performed an MLST analysis using partial sequences of four core housekeeping genes (16S rRNA, *gyr*B, *rpo*B and *rpo*D) of type strains of *Pseudomonas* [10,11] as well as close related *P. fluorescens* species. Cluster analysis and phylogenetic tree (Figure 1) showed that the tested ITEM strains can be reasonably placed within the subgroups formed by the *P. lactis* and *P. paralactis* species recently described by von Neubeck et al. [12].

Amending the previous description of *Pseudomonas* spp. ITEM 17298 [22,24], this isolate should be correctly classified as *Pseudomonas lactis*. Hereinafter, these bacteria will be referred to as *P. lactis*.

In Supplementary Tables S1 and S2, ANI and DDH matrices of *Pseudomonas* spp. Genome are reported. According to ANI analysis, the closest relatives for ITEM 17295 is *P. fluorescens* Ps_22, with 98.57% of ANI, while ITEM 17298 and ITEM 17299 share 100% of nucleotide identity and are highly similar to *P. fluorescens* 11293 and Ps_22 with 98.55 and 98.54% of ANI, respectively. The highest value of DDH for ITEM 17298 and ITEM 17299 is the reciprocal 99.8%, while the highest value of DDH for ITEM 17295 is towards *P. fluorescens* Ps_20 (89.5%).

**Figure 2:**
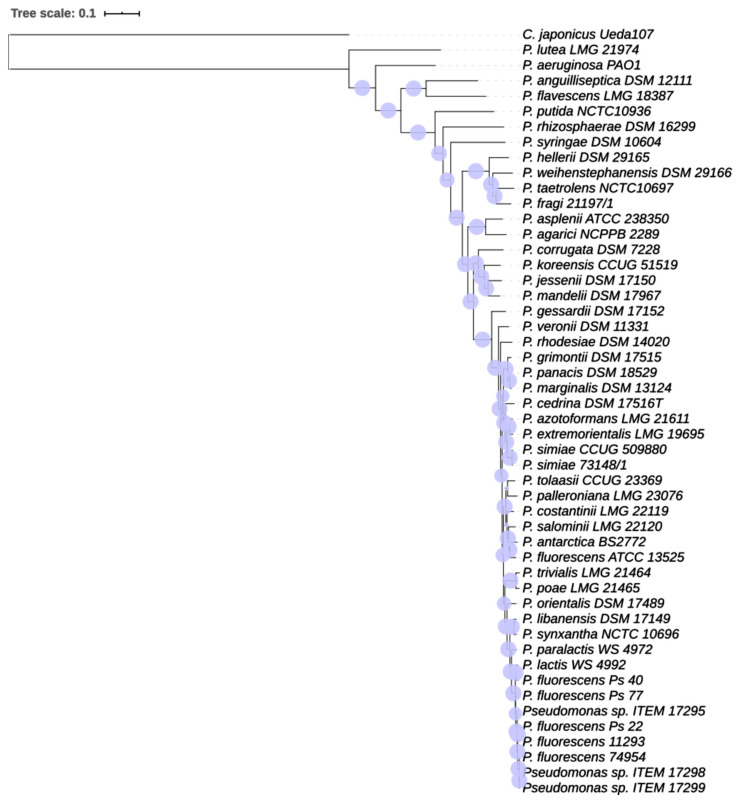
Genome-based phylogenetic tree inferred by using the Maximum Likelihood method RAxML with progressive refinement. *Celvibrio japonicus* Ueda 107 was used as outgroup. The tree is drawn to scale. Support values are represented by scaled circles at each node. Circles are representative of bootstrap values.

Both phylogenetic trees (Figure 1 and Figure 2) obtained by MLST analysis and Randomized Axelerated Maximum Likelihood (RAxML) analysis confirmed the clustering of *Pseudomonas* strains isolated from milk or dairy environment, validating the genetic similarity of the strains and the proposed novel species described by von Neubeck et al. [12].

According to Chun et al. [8], who defined genome-based criteria for the taxonomy of prokaryotes, we can assume that *Pseudomonas* sp. strain ITEM 17295, strain ITEM 17298, strain ITEM 17299, strain Ps_22, strain Ps_40, strain Ps_20, strain Ps_77, strain 11293, and strain 74954 should all be classified as *P. lactis*. In addition, although von Neubeck et al. [12] described *P. lactis* and *P. paralactis* as different species according phenotypic characteristics, they did not perform any phylogenetic tree based on core genome alignment. The analysis we performed (Figure 1 and Figure 2) clearly showed that all the strains cited above, with, additionally, *P. lactis* type strain WS 4992 and *P. paralactis* type strain WS 4672, are all included in the same paraphyletic clade, so they should be classified as the same species. The availability of a wider genomic dataset of *Pseudomonas* strains from dairy environment, with similar or different phenotypes, would help in the definition and resolution of this classification.

#### 3.1.3. Protein Functional Classification

More than 2400 UniProtKB AC/ID identifiers for each strain were successfully mapped to UniProtKB IDs (The UniProt Consortium, 2017). The retrieved list comprised about 60 genes involved in antibiotic resistance (coding for efflux pumps and multidrug transporters, beta-lactamase, fosmidomycin and bicyclomycin resistance protein 62, 65 and 60 genes for ITEM 17295, 17298 and 17299, respectively), more than 450 genes involved in transport (462, 497, 459), QS (*pvd*Q, *qui*P, *lux*R, *esa*R, *rpa*R, *bja*I in all strains; *ano*I and *ano*R in ITEM 17295 and 17299; *mqs*R is truncated in ITEM 17295) and hemolysis (*shl*B, *shl*A, *hp*D, *tly*C, *apx*lB, *lkt*A and *hly*A, coding for an hemolysis-type calcium binding protein, which is present only in ITEM 17298 and ITEM 17299).

Predicted genes were assigned to the clusters of orthologous groups (COG) classification (Supplementary Figure S1). Despite the group of general function prediction, which was the largest, the highest count for both strains was related to amino acid transport and metabolism and to inorganic ion transport and metabolism followed by signal transduction mechanisms.

**Figure 3:**
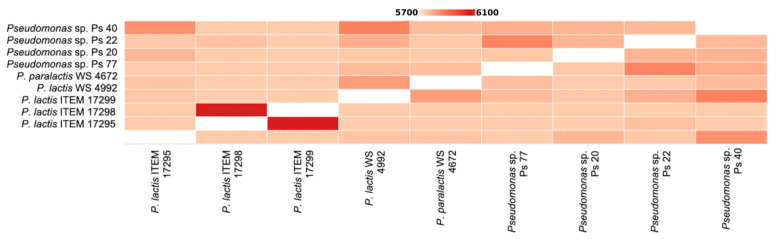
Pairwise heatmap of overlapping cluster between pairs of genomes.

Figure 3 shows the pairwise heatmap which visualizes the overlapping cluster numbers for nine *Pseudomonas* isolates. ITEM 17298 and ITEM 17299 shared the highest number of orthologue cluster (6055), followed by *Pseudomonas* spp. Ps_40 and *P. lactis* WS 4992 (5863) and *Pseudomonas* spp. Ps_77 and Ps_22 (5861). The species form 7004 clusters, 2882 orthologous clusters (at least contains two species), and 4122 single-copy gene clusters. All nine isolates shared 4171 clusters with a total of 37,689 proteins counted.

Belonging to the clusters unique to ITEM 17298, OrthoVenn2 analysis indicated two Phage-related minor tail proteins, and, not shared with the other ITEM strains or with *P. lactis* or *P. paralactis*, several hypothetical proteins with no GO (Gene Ontology) associated, one protein involved in response to arsenic-containing substance, phage related proteins, proteins for the viral genome integration into host DNA and conjugation proteins. The same occurs for ITEM 17295, which has a wide repertoire of viral tail assembly and integration proteins and, unique to all other isolates, two Epoxide hydrolase-like proteins (GIB65_13865 and GIB65_16720).

### 3.2. Phenotypic and Genomic Characterization

#### Biofilm and Motility

**Figure 4:**
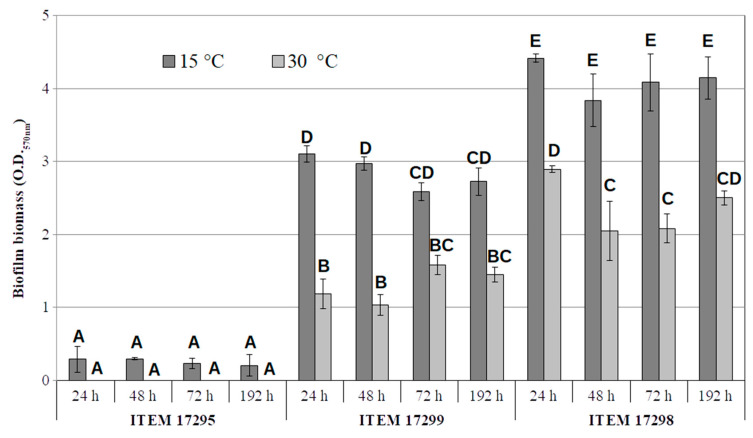
Biofilm biomass by *P. lactis* ITEM 17295, ITEM 17299, ITEM 17298 after 192 h at 15 and 30 °C. Bars are the average ± standard deviation (*n* = 3). Similar values (*p* > 0.05) are represented by the same subscript letters according to the HSD Tukey post-hoc test.

As reported in Figure 4, ITEM 17298 produced the highest amount of biofilm under all experimental conditions over the incubation time (Absorbance CV value > 4). In addition, ITEM 17299 was a strong biofilm producer; however, its amount was significantly (*p* < 0.05) lower by 30 and 43%, on average, than ITEM 17298 both at 15 and 30 °C, respectively. In accordance with our previous data [22], both strains doubled biofilm biomass at the lowest temperature. By contrast, ITEM 17295 showed no detectable biofilm biomass at 30 °C, whilst absorbance values lesser than 0.3 were registered at 15 °C over the entire period of incubation. Statistical analyses indicated that biofilm production significantly depended on the strains and temperature, whilst the incubation time was irrelevant; in addition, the interaction between time and temperature showed no significant (*p* > 0.005) effect on biofilm formation by each tested strain. Therefore, the low temperature was more effective than that of the optimal growth to promote biofilm production only for ITEM 17298 and 17299.

As widely reported, biofilms are organized structures surrounded by extracellular matrix in which the cells are embedded and protected from the environmental stresses. Recently, biofilms and QS have been suggested as important factors in the deterioration of dairy products, opening new fields of investigation and challenges to counteract spoilage [21,24,51,52]. In particular, the production of hydrolytic enzymes (extracellular proteases, lipases, glycosidases, and phospholipases) have been demonstrated to be enhanced in QS regulated-biofilms by several bacterial spoilers, including *P. fluorescens* [51,52,53]; thus, innovative antibacterial strategies based on the application of QS inhibitors have been considered promising to prevent food spoilage.

Among factors affecting the biofilm phenotype, a positive correlation between low temperatures and biofilm production was previously demonstrated in foodborne *P. fluorescens* [54,55]. In accordance with these authors, our results highlighted that low incubation temperatures were more important than incubation time for biofilm formation, putatively favoring spread and persistence in the dairy sector.

In addition to biofilm formation, *P. lactis* motility (swimming, swarming and twitching) was also investigated. Indeed, cell surface characteristics (occurrence of chemotaxis systems or hydrophobicity) and motility are crucial in nutrient acquisition, cell–cell and cell–surface interactions, stress tolerance, sites colonization and the initial attachment during biofilm formation [56]. As expected, patterns of motility were different depending on agar percentage used (Supplementary Figures S2 and S3); low agar percentage (0.3%) favored swimming motility (Supplementary Figure S2), a flagella-mediated movement in liquid media [57]; swimming motility was registered for each strain and mainly affected under higher temperature of incubation.

ANOVA analysis revealed that there was a statistically significant interaction between the effects of strain, time, and temperature on swarming (*F*(4, 36) = 3.719, *p* = 0.0012). In particular, swarming of all strains significantly increased as a time-dependent manner at the optimal growth temperature, whereas, at low temperatures, they swarmed later but to a lesser extent; ITEM 17295 showed smaller time-dependent increments at both incubation temperatures (Supplementary Figure S3). No appearance of elongated swarm edge was recorded over the time. Twitching motility, requiring type IV pili, was instead registered for ITEM 17298 at both temperature and significantly (*χ*^2^(5) = 52.89, *p* < 0.0001; Kruskal–Wallis rank sum test) increased depending on time of incubation reaching the highest value at 72 h; at the end of incubation, ITEM 17299 exhibited a twitching only at 15 °C but with an average value significantly lesser than those of ITEM 17299 (Supplementary Figure S3).

Based on these results, a genomic determinant related to the above reported phenotypic traits was identified. Table 2 listed most biofilm-related genes in all analyzed *P. lactis* strains; genes were catalogued with respect to their role in the consequential steps occurring during biofilm formation, including control mechanisms of motility apparatus. Even though results from biofilm and motility assay demonstrated a specific behavior for each strain, comparative analysis highlighted that all strains shared the majority of the biofilm-related genes. This is expected, considering the genetic similarity of the strains described in previous paragraphs. Differences occurred in a just few genes: among the most relevant, ITEM 17295 missed the *pel* operon, orthologue to the previously described in *P. aeruginosa* PA3058-PA3064. The operon codes for pellicle biofilm biosynthesis proteins, and it is present in ITEM 17298 (PROKKA_02829-PROKKA_02835) and ITEM 17299 (GIB64_23225-GIB64_23255) (Table 2). In *P. aeruginosa*, *pel* operon is involved in the formation on the extracellular matrix that surround the cells in a mature biofilm [58]. In addition, it modulates colony morphology giving rise to wrinkled red colonies on Congo red agar; these traits were confirmed by *pel* deletion in mutant strains of *P. aeruginosa* appearing as orange and invariably flat and smooth colonies [59]. Differences in color were correlated to the production or the absence of a Congo-red-binding component of the biofilm matrix. Although both ITEM 17298 and ITEM 17299 genomes harbor *pel* operon, a wrinkled red morphology appeared only in ITEM 17298 when incubated at 15 °C (Supplementary Figure S4), suggesting that *pel* operon genes are differently regulated in these strains and putatively expressed only in ITEM 17298 in this latter condition. As expected, orange and flat colonies were instead obtained for ITEM 17295 (Supplementary Figure S4).

Among the genes shared by all three ITEM strains, we found the alginate and *psl* operons; alginate operon showed a genetic organization similar but not identical to that of *P. aeruginosa*, in which *arn* operon is not contiguous to the *alg*D operon, as reported by Quintieri et al. [24].

In addition, we identified orthologue of the biofilm dispersion protein *bdl*A (PROKKA_05334, PROKKA_03805, GIB65_02045, GIB65_16355, GIB64_15510, GIB64_03410) the *pga*ABCD locus, which in *E. coli* promotes the synthesis of a polysaccharide adhesin required for biofilm formation [60] and fimbrial proteins (PROKKA_00968, GIB65_15860, GIB64_11150).

As shown in Table 2, differences among assayed strains were found for Toxin–Antitoxin (TA) systems (ChpB/ChpS; MqsR/MqsA); these latter were also identified in several *Pseudomonas* spp. genomes [61,62,63].

Although their role in cell physiology needs to be investigated further, some TAs are involved in the switch from the planktonic to the biofilm lifestyle, as well as virulence and multidrug resistance in pathogenic pseudomonads [61,62,63]; in particular, MqsR/MqsA plays an important regulatory role in the persistence and biofilm formation by *P. putida* and *E. coli* [61]. Few differences were also retrieved in the content of biofilm related genes in the genomes of other *P. lactis*, such as *P. lactis* SS101 and *P. lactis* DSM 29,167 (Fanelli, unpublished results), highlighting certain genetic variability among these strains.

As reported in Supplementary Table S3, no differences were found among strains in the list of genes involved in flagella biosynthesis and twitching motility. This list was obtained by homology search towards *P. aeruginosa*, possessing a single polar flagellum that plays a variety of roles in the virulence in addition to its central role in swimming motility. In all strains, we retrieved quite the same genetic content and organization of *P. aeruginosa*, with genes encoding the flagellar basal body MS ring and motor switch complex, and genes coding for flagella assembly and the sigma factor regulating this process *fli*A and *rpo*N [64,65], as well as the *fle*QSR locus coding for the major flagella gene regulator in *P. aeruginosa* and the two-component sensor system *fle*SR involved in flagellin synthesis. By contrast, the flagellar glycosyltransferase FgtA, involved in the *P. aeruginosa* flagellin glycosylation that in turn exacerbates its virulence [66], was absent in all *P. lactis.* Twitching motility genes, such as *pil*T, *pil*G, *pil*H, *pil*I, and *pil*J were also identified.

### 3.3. Pigment Production

*Pseudomonas* spp. strains are able to release multifunctional pigments. Although in dairy products they are primarily implicated in spoilage [17], in the bacterial cells they exhibit specific activities including regulatory functions [67] and protective roles under stress response [68]. In addition, for this phenotypic trait, each strain showed a specific behavior (Supplementary Table S4); no pigments were released on the selective media by ITEM 17295, whilst pigmented colonies were found for the remaining strains (Supplementary Table S4). The list of identified genes referred to pyoverdines, pyomelanin, and indigo-derivatives synthesis is shown in Supplementary Table S5.

As concern pyoverdines, genes are shared and contiguous, and similarly organized in all the ITEM strains, with the only exception of *pvd*S, coding for a sigma factor which is required for the expression of pyoverdine biosynthetic genes [69], in contrast to ITEM 17298 and 17299; indeed, this latter was predicted as a pseudogene in ITEM 17295. This could explain in ITEM 17295 the absence of pyoverdine on the selective medium. In previous studies, it has been revealed that *pvd*S encodes an alternative sigma factor which directs RNA polymerase to the promoters of pyoverdine biosynthesis genes; mutants of *pvd*S gene in *P. aeruginosa*, indeed, failed to make detectable pyoverdine when grown on King’s B agar or in liquid medium [70].

Among the relevant differences we identified among our three *P. lactis* strains are the absence in ITEM 17295 of the *trpC* gene coding for the tryptophan biosynthesis protein (PROKKA_00895, GIB64_11525) and the tryptophan synthase alpha and beta chain (PROKKA_00904-00905; GIB64_11475-11480), which were demonstrated to be involved in the pathways leading to the formation of indigo-derivatives.

The locus comprising these genes is homologue to the c4_BAR (Contig 4 Blue Accessory Region) that Andreani et al. [71] identified as only present in Blue pigmenting pseudomonads strains. This region was described as containing 16 genes (16 Kb) including those coding for *trp* accessory genes: *trp*D, *trp*F, *trp*A, and *trp*C.

A deeper investigation into our pigmenting strains ITEM 17298 and ITEM 17299 confirmed the presence of this locus in both strains, a locus that is absent in ITEM 17295 that is not pigmenting; the genomic context of the flanking regions (data not shown) reveals the occurrence of genetic elements which suggest the presence of integrative conjugative plasmid. Indeed, we identified several integrating conjugative elements, as well as pilus formation and assembly proteins. Furthermore, we detected genes coding for one integrase as well as a locus comprising several t-RNA coding genes, which are a hotspot for genomic island integration.

### 3.4. Protease and Lipase

**Figure 5:**
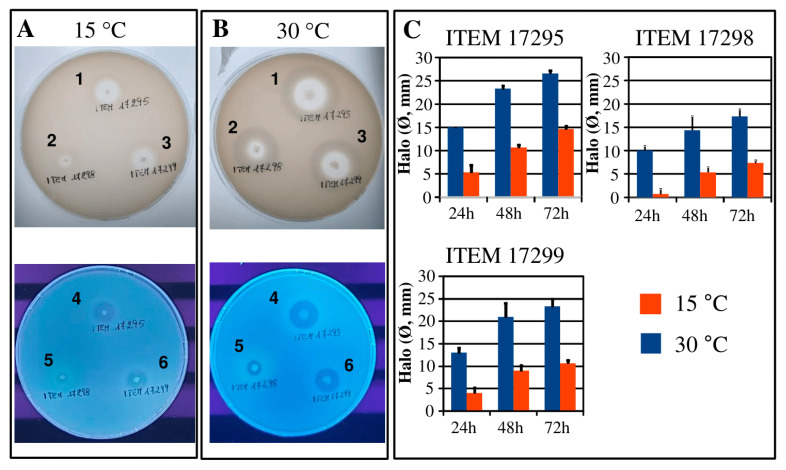
Halos of proteolytic activity produced by *Pseudomonas lactis* ITEM 17295 (A1, B1, A4, B4), ITEM 17298 (A2, B2, A5, B5), and ITEM 17299 (A3, B3, A6, B6) patched onto milk agar plates, incubated at 15 and 30 °C for 72 h and revealed under visible light (**A**,**B**, upper side) and related UV lamp fluorescence (panels **A**,**B**, lower side). Bars in the graphs represent diameter means (±standard deviation, *n* = 3) of clear proteolytic halos registered at 24, 48, and 72 h (**C**).

The tested strains exhibited hydrolytic activity against milk proteins showing a time-dependent manner increase of proteolytic activity as the following order: ITEM 17295 > ITEM 17299 > ITEM 17298 (Figure 5); these results agreed with our previous results describing ITEM 17295 (called PS37) as strong proteolytic strain in milk samples [12]. The incubation at the temperature lower than that of optimal growth caused a significant decrease in halo diameters, much greater for ITEM 17298 (Figure 5).

The genomic analysis identified several protease genes (*apr*A, *prs*DE, *prt*AB) in all tested strains (Supplementary Table S6). These genes are positioned in a QS-regulated operon previously associated with milk spoilage by *P. fluorescens* [72]. In particular, *apr*A, encoding a heat stable caseinolytic alkaline metalloprotease [73], is widespread in *Pseudomonas*, especially in the psychrotrophic strains [74] to the extent that it is recommended in the search of milk spoilage pseudomonads instead of *rpo*B [75]. In previous works, AprA isolated from several *Pseudomonas* spp. was described as 98% similar to the peptidase AprX secreted by one strain of *P. fluorescens* [72]. AprA and AprX generally exhibit activity in a large range of temperatures (0–55 °C) with optimal activity between 37 and 47 °C [76]. In addition to the improved growth at 30 °C, this could explain the diameter increase registered in all strains under this experimental condition (Figure 5).

Although all the herein assayed strains shared the spoilage *aprA*-*lipA* operon, the genome of the most proteolytic strain, ITEM 17295, bears a second gene coding for a metallopeptidase AprA (family M10) GIB65_09350 (Supplementary Table S7). This genomic locus comprising *apr*A also included the gene coding for chemotaxis protein CheB, protein-glutamate O-methyltransferase CheR, several response regulators, transporters, and several fimbrial proteins, and is flanked by two recombinase/integrase. A similar locus was retrieved also in the other strains, leading to the assumption that this locus is derived from an integration event, but with relevant differences in the gene content, with *apr*A and other elements exclusive of ITEM 17295.

Supplementary Table S7 reports a list of the peptidases/transpeptidases identified in each *P. lactis* genome; beyond proteolytic activity, several enzymes were involved in biological processes, such as stress response and signal transduction. In accordance with the high similarity among strains, just a few differences were revealed.

The examination of *P. lactis* proteolytic halos under UV lamp revealed fluorescence around ITEM 17928 colonies, putatively due to pyoverdine release (Figure 5, panel B, lower side) and most marked with regard to the growth at low temperature. These results agreed with the results reported above for pigment release. Indeed, only ITEM 17298 produced pyoverdine although in synthetic media the synthesis mainly occurred at the low temperature. As widely reported in literature, pyoverdines can be considered as signaling molecules mediating specific mechanisms as the spoilage induced by proteases and lipases activities [77,78,79]. Preservation techniques, indeed, modulating pyoverdines synthesis to counteract pseudomonads growth, spoilage, or pathogenesis are a current challenge [80].

Lipase activity was apparently registered after 72 h of incubation only for the strains ITEM 17295 and ITEM 17299 colonies displaying under UV lamp a brilliant glow pink-red color and putatively associated with intracellular release of fatty acids (Supplementary Figure S5). At a low temperature, only ITEM 17295 proved to grow on the Rhodamine agar with fluorescent smaller colonies (Supplementary Figure S5). However, evident blue fluorescent halos were registered around colonies of all tested strains grown at 30 °C putatively associated with the pyoverdine release. As reported above, two lipases were identified in the spoilage operon (Supplementary Table S6) in all assayed strains. The activity of lipase included in *apr*X-*lip*A operon are regulated by EnvZ-Ompr [60], herein identified and annotated as PROKKA_02877-PROKKA_02878 for ITEM 17298, GIB65_26250-GIB65_26255 for ITEM 17295, and GIB64_23460-GIB64_23465 for ITEM 17299. Although each strain exhibited different lipolytic activity, no differences were reported in the list of lipolytic enzymes by genomic analysis (Supplementary Table S8); this suggested that, in each *P. lactis* strain, different regulatory mechanisms could activate the expression of these genes.

## 4. Conclusions

In this study, we analyzed three cheese-borne spoilage *Pseudomonas* in order to identify the genomic determinants responsible for their phenotypic traits. Based on the results of validated genomic methods, these strains grouped in the paraphyletic clade of *Pseudomonas lactis*, and thus they were classified as so. Despite their high genetic similarity, phenotypic analysis showed distinctive traits in relation to biofilm formation, pigmentation, proteolytic, and lipolytic activities. Some of these characteristics (biofilm and pigmentation) are promoted at lower temperatures of incubation, suggesting their possible involvement in the spreading and persistence of these strains in the dairy environment. Several differences in the identified genetic elements were consistent with the phenotypic diversity among these strains. In this work, psychrotrophic pseudomonad strains spoiling mozzarella cheese were reclassified as *P. lactis*, previously associated with the spoilage of the refrigerated raw milk. The availability of two novel *P. lactis* genomic sequences and the analysis here performed will improve the knowledge about the genetic elements involved in their spread, persistence, and spoilage, thus paving the way towards novel preservative strategies.

## Figures and Tables

**Table 1:** Genome features of *Pseudomonas* sp. strains.

Features	ITEM 17295	ITEM 17298	ITEM 17299
Genome size (bp)	6,288,321	6,288,436	6,244,929
GC (%)	59.83	59.75	59.78
Number of contigs	89	117	137
Completeness	93.7%	93.7%	93.7%
Quality	89.2 (excellent)	89.2 (excellent)	89.2 (excellent)
Contig N50	351,021	146,994	139,064
Genes (total)	5736	5910	5752
Genes (coding)	5595	5734	5617
Coding density	88.81%	88.60%	88.55%
rRNAs	2, 1, 1 (5S, 16S, 23S)	3, 1, 1 (5S, 16S, 23S)	2, 1, 1 (5S, 16S, 23S)
tRNAs	68	55	54

**Table 2:** Main biofilm related proteins in *P. lactis* strains.

Biofilm Formation Step		Genes	ITEM 17295	ITEM 17298	ITEM 17299
**Attachment**	*Switching from planktonic to biofilm state*	*csr*A/*rsm*A	GIB65_24765	PROKKA_01891	GIB64_12670
		c-di-GMP regulated *gac*S/*gac*A	GIB65_18615/ GIB65_01605	PROKKA_02681/ PROKKA_01410	GIB64_19800 GIB64_28045
		*lux*Q	GIB65_08270	PROKKA_00312	GIB64_13935
		*luxR*	GIB65_04815	PROKKA_03671	GIB64_14810
		*rhlR/rhlI*	GIB65_06785/ GIB65_06780	PROKKA_01310/ PROKKA_01311	GIB64_11890/ GIB64_11895
		*sag*S	GIB65_08650	PROKKA_03896	GIB64_26065
		*hpt*B	GIB65_07985	PROKKA_01759	GIB64_17280
		*hsb*R-*hsb*A	GIB65_07985- GIB65_07990	PROKKA_01760- PROKKA_01761	GIB64_17280- GIB64_17285
		*lad*S/*ret*S	GIB65_05330/ GIB65_20530	PROKKA_03621/ PROKKA_03260	GIB64_14560/ GIB64_03985
		c-di-GMP regulated *bif*A	GIB65_02210	PROKKA_05637	GIB64_07760
		cAMP Phosphodiesterase *cdp*A	GIB65_19990	PROKKA_03152	GIB64_21855
		*rbd*A	GIB65_07415	PROKKA_01243	GIB64_00920
		*yda*M	GIB65_04035	PROKKA_01515	GIB64_25020
		*yfi*B	GIB65_04455	PROKKA_01205	GIB64_26920
		c-di-GMP regulated *tpb*B-*yfi*N	GIB65_04460	PROKKA_01204	GIB64_26915
		*yfi*R	GIB65_04465	PROKKA_01203	GIB64_26910
		*mqs*R/*mqs*A	pseudo */ GIB65_14795	PROKKA_01685/ PROKKA_01686	GIB64_02008/ GIB64_02009
		*chpB/chpS*	NA	PROKKA_00869/ PROKKA_00870	GIB64_11655/ GIB64_11650
	*Pilus biosynthesis and twitching motility*	*pil*z domain-containing protein	GIB65_04785	PROKKA_03677	GIB64_14840
		*pil*GHIJ	GIB65_22310 GIB65_22295	PROKKA_00456- PROKKA_00459	GIB64_13350 GIB64_13365
		*pil*A	GIB64_11150	PROKKA_00968	GIB65_15860
	*Flagellar gene expression (swimming, swarming motility)*	*fle*Q	GIB65_08040	PROKKA_01770	GIB64_17225
		*fli*A	GIB65_07910	PROKKA_01745	GIB64_17355
		*flg*M	GIB65_27400	PROKKA_05108	GIB64_24150
	*Microbial cell adhesion and/or swarming motility*	*rhl*A	GIB65_22015	PROKKA_02434	GIB64_28365
		*pvd*Q	GIB65_09880	PROKKA_00251	GIB64_05820
		*ple*D	GIB65_20585	PROKKA_03271	GIB64_04040
		cyclic-Di-GMP phosphodiesterase *bif*A	GIB65_02210	PROKKA_05637	GIB64_07760
	*Adhesion*	*pga*ABCD	GIB65_26770 GIB65_26785	PROKKA_04557 PROKKA_04560	GIB64_08340 GIB64_08355
	*Chemotaxis*	*che*A/*che*W	GIB65_22290/ GIB65_22295	PROKKA_00460/ PROKKA_00461	GIB64_13370/ GIB64_13375
		*che*BAZY	GIB65_07890 GIB65_07905	PROKKA_01741 PROKKA_01744	GIB64_17375 GIB64_17360
		*wsp*RFEDCBA	GIB65_13580 GIB65_13610	PROKKA_04814 PROKKA_04820	GIB64_04640- GIB64_04610
	*SurfaceSensing*	cAMP-*vfr*	GIB65_21375	PROKKA_04713	GIB64_01775
**Microcolonies formation and maturation**	*QS-signals*	*trp*E/*trp*G	GIB65_21405/ GIB65_21395	PROKKA_04707/ PROKKA_04709	GIB64_01805/ GIB64_01795
		*fab*G	GIB65_27840	PROKKA_02061	GIB64_16215
		*ppk*A	GIB65_23520	PROKKA_03312	GIB64_09480
	*Cell-cell signals*	type VI secretion system T6SS	GIB65_23600 GIB65_23520	PROKKA_03296 PROKKA_03311	GIB64_09560 GIB64_09480
		type VI secretion system protein TssB	GIB65_19080 GIB65_19150	PROKKA_04236 PROKKA_04222	GIB64_25965 GIB64_25895
		*bfm*R/*bfm*S	GIB65_09040/ GIB65_09045	PROKKA_00299/ PROKKA_00300	GIB64_14000/ NA
	*Eps formation*	*psl*ABCDEFGHIJKL	GIB65_16955 GIB65_16900	PROKKA_04436 PROKKA_04447	GIB64_17790 GIB64_17845
		*pel*ABCDEFG	NA ^1^	PROKKA_02829- PROKKA_02835	GIB64_21390- GIB64_21420
	*Alginatebiosynthesis*	*muc*R	GIB65_00065	PROKKA_05333	GIB64_26185
		*alg*D operon	GIB65_14860- GIB65_14920	PROKKA_01700- PROKKA_01712	GIB64_10145- GIB64_10205
**Detachment**	*chemotaxis*	Biofilm dispersion protein BdlA	GIB65_02045, GIB65_16355	PROKKA_03805, PROKKA_05334	GIB64_15510, GIB64_03410
		Phytochrome-like protein *cph*2	GIB65_12695	PROKKA_00099	GIB64_12320

^1^ NA: not annotated; *: pseudogene.

## Supplementary Materials

The following are available online at https://www.mdpi.com/2076-2607/8/8/1208/s1. Figure S1. COGs functional classification of genes present in *P. lactis* ITEM 17295, ITEM 17298, and ITEM 17299 genomes. Figure S2. Swimming motility of *P. lactis* ITEM 17295, ITEM 17298 and ITEM 17299, in LB performed at 15 °C and 30 °C for 24, 48 and 72 h. Bars represent the mean diameters (±standard deviation, *n* = 3) of corresponding motility zones. Similar values (*p* > 0.05) are represented by the same subscript letters according HSD Tukey post-hoc test. Figure S3. Swarming and twitching motility of *P. lactis* ITEM 17295, ITEM 17298 and ITEM 17299, registered in LB and M8 medium, respectively for 24, 48, and 72 h at 15 °C and 30 °C. Bars represent the mean diameter (±standard deviation, *n* = 3) of corresponding motility zones. Twitching motility values were determined by measuring the absorbance of Crystal violet (CV) at 570 nm. Bars represent the average ± standard deviation (*n* = 3). Similar values (*p* > 0.05) of swarming and twitching are represented by the same subscript letters according HSD Tukey post-hoc test and Wilcoxon rank sum test and *p*-value adjustment Bonferroni’s method, respectively. Figure S4. Colony morphologies of *P. lactis* ITEM 17295, ITEM 17298, and ITEM 17299 on Congo Red agar plates containing 1.5% of agar (panel A), and LB agar plates, containing 0.3% of agar and Congo Red dye (swimming assay; panel B), after 72 h of incubation at 15 °C and 30 °C (panel B). Figure S5. Lipolytic activity of *P. lactis* ITEM 17295, ITEM 17298 and ITEM 17298 strains patched on Rhodamine agar plates, incubated for 72 h at 15 and 30 °C and revealed under UV lamp as fluorescent glow pink-red colonies. Bars represent the average (±standard deviation, *n* = 3) of registered fluorescent colony diameters. Table S1. ANI matrix of *Pseudomonas* spp. strains. Table S2. DDH matrix of *Pseudomonas* spp. strains. Table S3. List of genes coding for proteins involved in flagella biosynthesis and twitching motility. Table S4. Colony appearance of *P. lactis* ITEM 17295, ITEM 17298, and ITEM 17299 grown on selective media (King A, King B, PDA) for 5 days under 15 and 30 °C. Table S5. List of gene coding for protein involved in pigment metabolism in *P. lactis* strains. Table S6. Spoilage operon in *P. lactis* strains. Table S7. Proteases identified in *P. lactis* strains. Table S8. Lipases identified in *P. lactis* strains.

## Abbreviations

MLST	Multilocus Sequence Typing
COG	Clusters of Orthologous Groups
CV	Crystal Violet
EPS	Extracellular Polymeric Substances
QS	*Quorum Sensing*
TA	Toxin–Antitoxin
RAxML	Randomized Axelerated Maximum Likelihood
GO	Gene Ontology
