# Supplementary material for: Genomic Analysis of Three Cheese-Borne Pseudomonas lactis with Biofilm and Spoilage-Associated Behavior
Source: Microorganisms. 2020 Aug 8;8(8):1208. doi: 10.3390/microorganisms8081208 (PMC7464908; doi:10.3390/microorganisms8081208)
Supplement: Supplementary file 1 [file microorganisms-08-01208-s001.zip › Supplementary materials/Table S3.docx]

| **Table S3. Flagellar and motility protein in *Pseudomonas* sp. strains** | | | | | |
| --- | --- | --- | --- | --- | --- |
| ***P. aeruginosa* PAO1** | | | ***P. lactis*** | | |
| **Locus Tag** | **Product** | **Protein ID** | **ITEM 17298** | **ITEM 17295** | **ITEM 17299** |
| PA1077 | flagellar basal-body rod protein FlgB | [NP_249768.1](http://www.ncbi.nlm.nih.gov/protein/NP_249768.1) | PROKKA_05104 | GIB65_27420 | GIB64_24130 |
| PA1078 | flagellar basal-body rod protein FlgC | [NP_249769.1](http://www.ncbi.nlm.nih.gov/protein/NP_249768.1) | PROKKA_05103 | GIB65_27425 | GIB64_24125 |
| PA1079 | flagellar basal-body rod modification protein FlgD | NP_249770.1 | PROKKA_05102 | GIB65_27430 | GIB64_24120 |
| PA1080 | flagellar hook protein FlgE | NP_249771.1 | PROKKA_05101 | GIB65_27435 | GIB64_24115 |
| PA1081 | flagellar basal-body rod protein FlgF | NP_249772.1 | PROKKA_01783 | GIB65_08105 | GIB64_17160 |
| PA1082 | flagellar basal-body rod protein FlgG | NP_249773.1 | PROKKA_01782 | GIB65_08100 | GIB64_17165 |
| PA1083 | flagellar L-ring protein precursor FlgH | NP_249774.1 | PROKKA_01781 | GIB65_08095 | GIB64_17170 |
| PA1084 | flagellar P-ring protein precursor FlgI | NP_249775.1 | PROKKA_01780 | GIB65_08090 | GIB64_17175 |
| PA1085 | flagellar protein FlgJ | NP_249776.1 | PROKKA_01779 | GIB65_08085 | GIB64_17180 |
| PA1086 | flagellar hook-associated protein 1 FlgK | NP_249777.1 | PROKKA_01778 | GIB65_08080 | GIB64_17185 |
| PA1087 | flagellar hook-associated protein type 3 FlgL | NP_249778.1 | PROKKA_01777 | GIB65_08075 | GIB64_17190 |
| PA1092 | flagellin type B fliC | NP_249783.1 | PROKKA_01775 | GIB65_08065 | GIB64_17200 |
| PA1094 | flagellar capping protein FliD | NP_249785.1 | PROKKA_01773 | GIB65_08055 | GIB64_17210 |
| PA1097 | transcriptional regulator FleQ | NP_249788.1 | PROKKA_01770 | GIB65_08040 | GIB64_17225 |
| PA1098 | two component sensor FleS | NP_249789.1 | PROKKA_01769 | GIB65_08035 | GIB64_17230 |
| PA1099 | two component sensor regulator FleR | NP_249790.1 | PROKKA_01768 | GIB65_08030 | GIB64_17235 |
| PA1100 | flagellar hook-basal body complex protein FliE | NP_249791.1 | PROKKA_01767 | GIB65_08025 | GIB64_17240 |
| PA1101 | Flagella M-ring outer membrane protein precursor | NP_249792.1 | PROKKA_01766 | GIB65_08020 | GIB64_17245 |
| PA1102 | flagellar motor switch protein FliG | NP_249793.1 | PROKKA_01765 | GIB65_08015 | GIB64_17250 |
| PA1103 | probable flagellar assembly protein | NP_249794.1 | PROKKA_01764 | GIB65_08010 | GIB64_17255 |
| PA1104 | flagellum-specific ATP synthase FliI | NP_249795.1 | PROKKA_01763 | GIB65_08005 | GIB64_17260 |
| PA1105 | flagellar protein FliJ | NP_249796.1 | PROKKA_01762 | GIB65_08000 | GIB64_17265 |
| PA1440 | hypothetical protein | NP_250131.1 | PROKKA_01795 | GIB65_08165 | GIB64_17100 |
| PA1441 | putative flagellar hook-length control protein FliK | NP_250132.1 | PROKKA_01758 | GIB65_07980 | GIB64_17285 |
| PA1442 | conserved hypothetical protein | NP_250133.1 | PROKKA_01757 | GIB65_07975 | GIB64_17290 |
| PA1443 | flagellar motor switch protein FliM | NP_250134.1 | PROKKA_01756 | GIB65_07970 | GIB64_17295 |
| PA1444 | flagellar motor switch protein FliN | NP_250135.1 | PROKKA_01755 | GIB65_07965 | GIB64_17300 |
| PA1445 | flagellar protein FliO | NP_250136.1 | PROKKA_01754 | GIB65_07960 | GIB64_17305 |
| PA1446 | flagellar biosynthetic protein FliP | NP_250137.1 | PROKKA_01753 | GIB65_07955 | GIB64_17310 |
| PA1447 | flagellar biosynthetic protein FliQ | NP_250138.1 | PROKKA_01752 | GIB65_07950 | GIB64_17315 |
| PA1448 | flagellar biosynthetic protein FliR | NP_250139.1 | PROKKA_01751 | GIB65_07945 | GIB64_17320 |
| PA1449 | flagellar biosynthetic protein FlhB | NP_250140.1 | PROKKA_01750 | GIB65_07940 | GIB64_17325 |
| PA1452 | flagellar biosynthesis protein FlhA | NP_250143.1 | PROKKA_01748 | GIB65_07925 | GIB64_17340 |
| PA1453 | flagellar biosynthesis protein FlhF | NP_250144.1 | PROKKA_01747 | GIB65_07920 | GIB64_17345 |
| PA1454 | flagellar synthesis regulator FleN | NP_250145.1 | NA* | GIB65_07915 | GIB64_17350 |
| PA1455 | Sigma factor FliA | NP_250146.1 | PROKKA_01745 | GIB65_07910 | GIB64_17380 |
| PA1460 | MotC | NP_250151.1 | PROKKA_01740 | GIB65_07885 | GIB64_17380 |
| PA1461 | MotD | NP_250152.1 | PROKKA_01738 | GIB65_07880 | GIB64_21920 |
| PA4953 | chemotaxis protein MotB | NP_253640.1 | [PROKKA_03165](https://blast.ncbi.nlm.nih.gov/Blast.cgi#alnHdr_Query_40259) | GIB65_20055 | GIB64_21915 |
| PA4954 | chemotaxis protein MotA | NP_253641.1 | PROKKA_03164 | GIB65_20050 | GIB64_17325 |
| PA0395 | twitching motility protein PilT | NP_249086.1 | [PROKKA_00446](https://blast.ncbi.nlm.nih.gov/Blast.cgi#alnHdr_Query_32559) | GIB65_22360 | GIB64_13300 |
| PA0396 | twitching motility protein PilU | NP_249087.1 | NA | NA | NA |
| PA0408 | twitching motility protein PilG | NP_249099.1 | [PROKKA_00456](https://blast.ncbi.nlm.nih.gov/Blast.cgi#alnHdr_Query_32569) | GIB65_22310 | GIB64_13350 |
| PA0409 | twitching motility protein PilH | NP_249100.1 | [PROKKA_00457](https://blast.ncbi.nlm.nih.gov/Blast.cgi#alnHdr_Query_32570) | GIB65_22305 | GIB64_13355 |
| PA0410 | twitching motility protein PilI | NP_249101.1 | [PROKKA_00458](https://blast.ncbi.nlm.nih.gov/Blast.cgi#alnHdr_Query_32571) | GIB65_22300 | GIB64_13360 |
| PA0411 | twitching motility protein PilJ | NP_249102.1 | [PROKKA_00459](https://blast.ncbi.nlm.nih.gov/Blast.cgi#alnHdr_Query_32572) | GIB65_22295 | GIB64_13365 |
| PA4462 | rpoN | NP_253152.1 | [PROKKA_01058](http://www.ncbi.nlm.nih.gov/protein/NP_253152.1) | GIB65_15405 | GIB64_10690 |
| NA = Not Annotated  * not annotated by PROKKA but manually annotated | | | | | |
