# Supplementary material for: Genomic Analysis of Three Cheese-Borne Pseudomonas lactis with Biofilm and Spoilage-Associated Behavior
Source: Microorganisms. 2020 Aug 8;8(8):1208. doi: 10.3390/microorganisms8081208 (PMC7464908; doi:10.3390/microorganisms8081208)
Supplement: Supplementary file 1 [file microorganisms-08-01208-s001.zip › Supplementary materials/Table S4.docx]

**Table S4.** Colony appearance of *P. lactis* strains grown on selective media (King A, King B and PDA) for 5 days at 15 and 30 °C.

| **Species** | **Strain** | **KING A** | | **KING B** | | **PDA** | |
| --- | --- | --- | --- | --- | --- | --- | --- |
|  |  | **15 °C** | **30 °C** | **15 °C** | **30 °C** | **15 °C** | **30 °C** |
| *P. lactis* | ITEM 17295 | C | C | C | C | C | C |
|  | ITEM 17299 | B | B | B | B | D | C |
|  | ITEM 17298 | B | C | B | F | D | C |

*F, green fluorescent; C, cream; B, brown; O, orange; Y, yellow; D, Dark
