# Supplementary figures and images for: Genomic Analysis of Three Cheese-Borne Pseudomonas lactis with Biofilm and Spoilage-Associated Behavior
Source: Microorganisms. 2020 Aug 8;8(8):1208. doi: 10.3390/microorganisms8081208 (PMC7464908; doi:10.3390/microorganisms8081208)

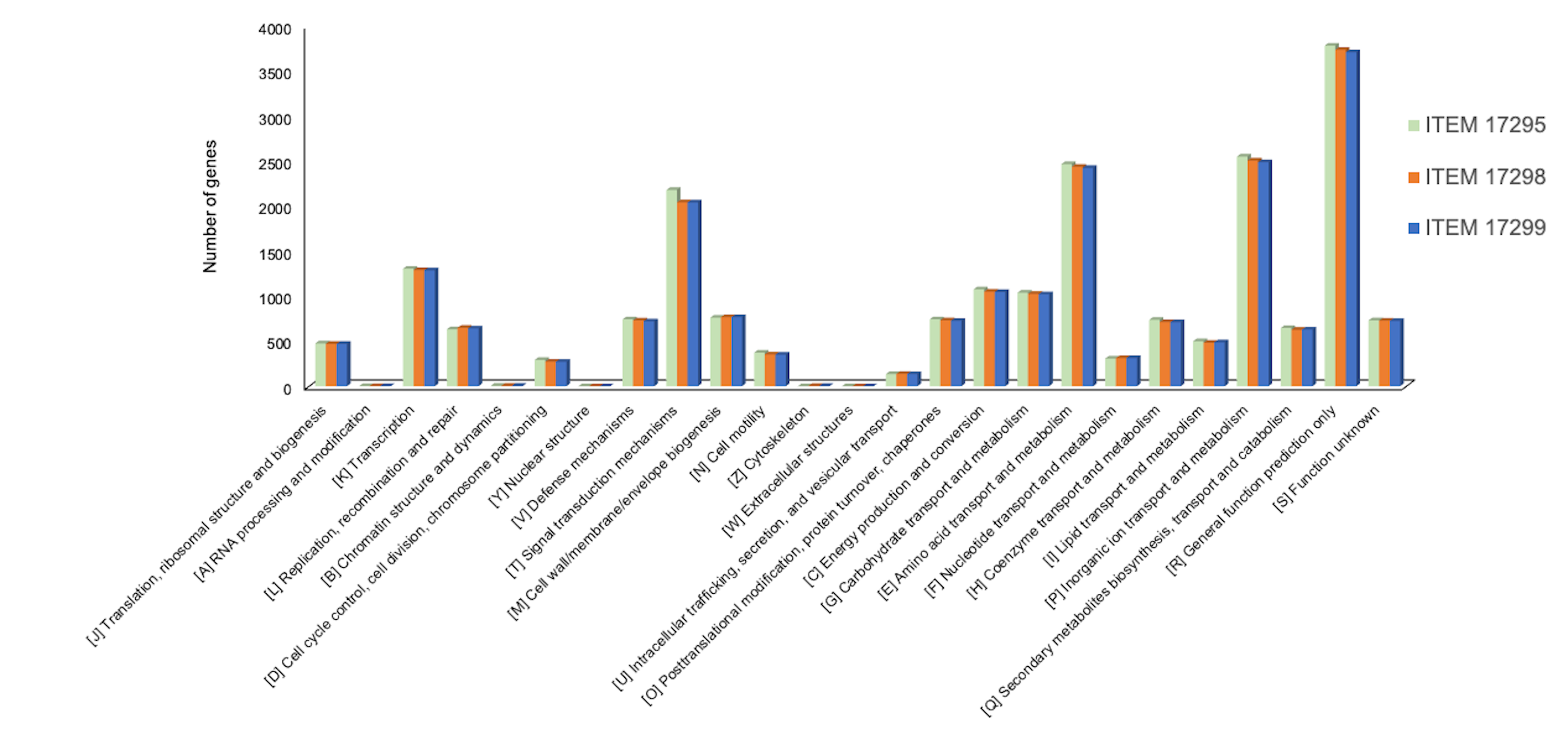

Supplement: Supplementary file 1 [file microorganisms-08-01208-s001.zip › Supplementary materials/Figure S1.png]

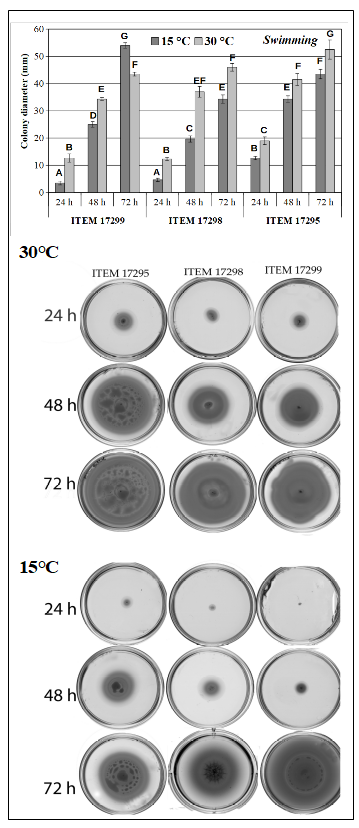

Supplement: Supplementary file 1 [file microorganisms-08-01208-s001.zip › Supplementary materials/Figure S2.png]

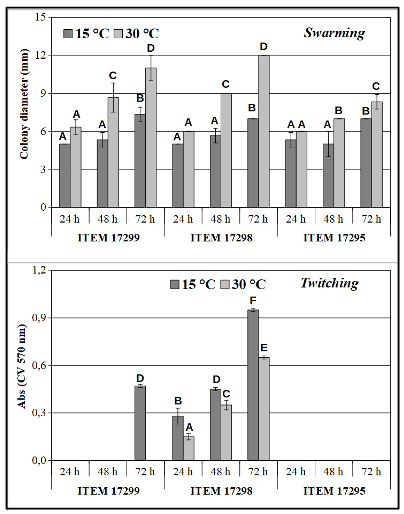

Supplement: Supplementary file 1 [file microorganisms-08-01208-s001.zip › Supplementary materials/Figure S3.png]

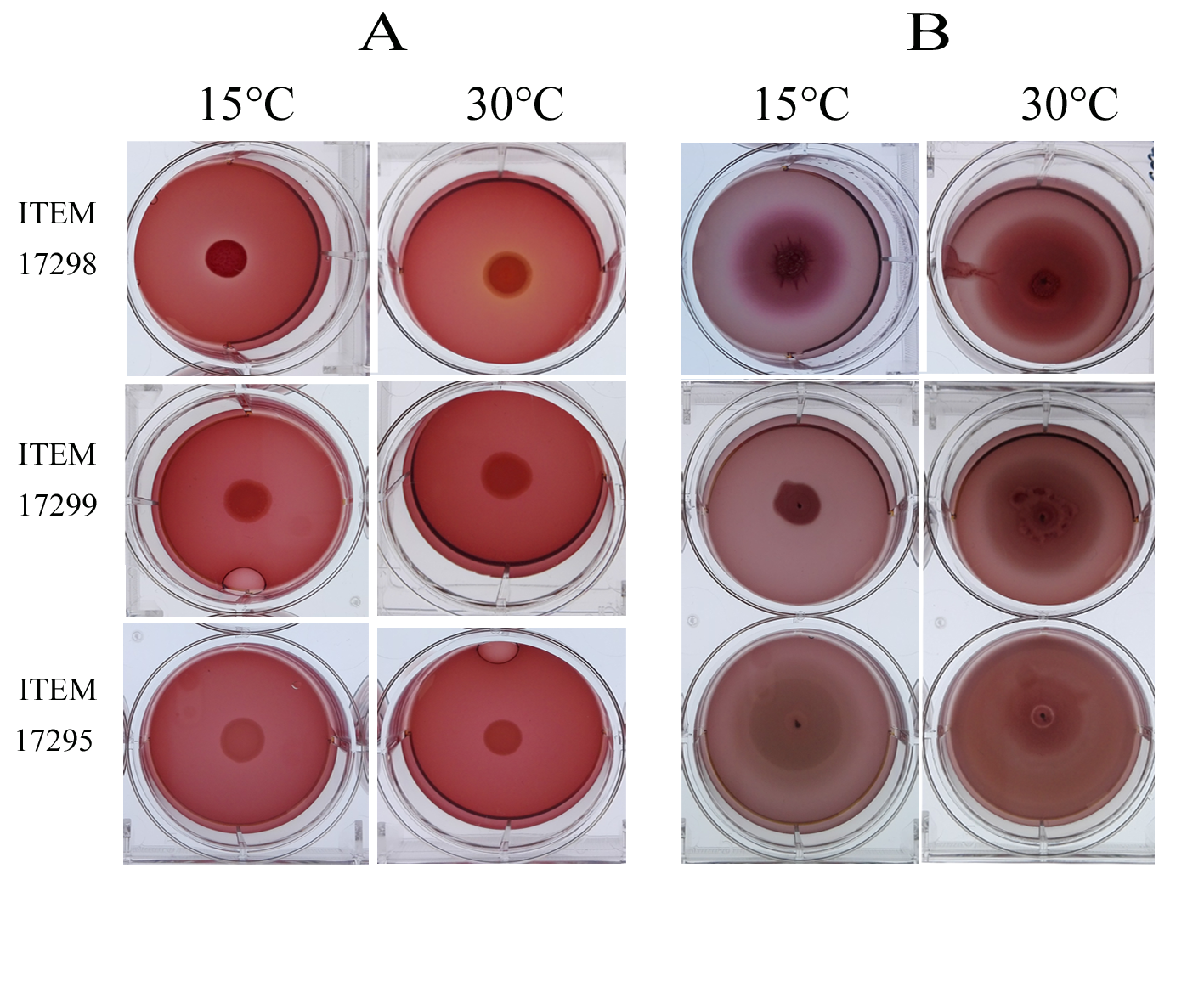

Supplement: Supplementary file 1 [file microorganisms-08-01208-s001.zip › Supplementary materials/Figure S4.tif]

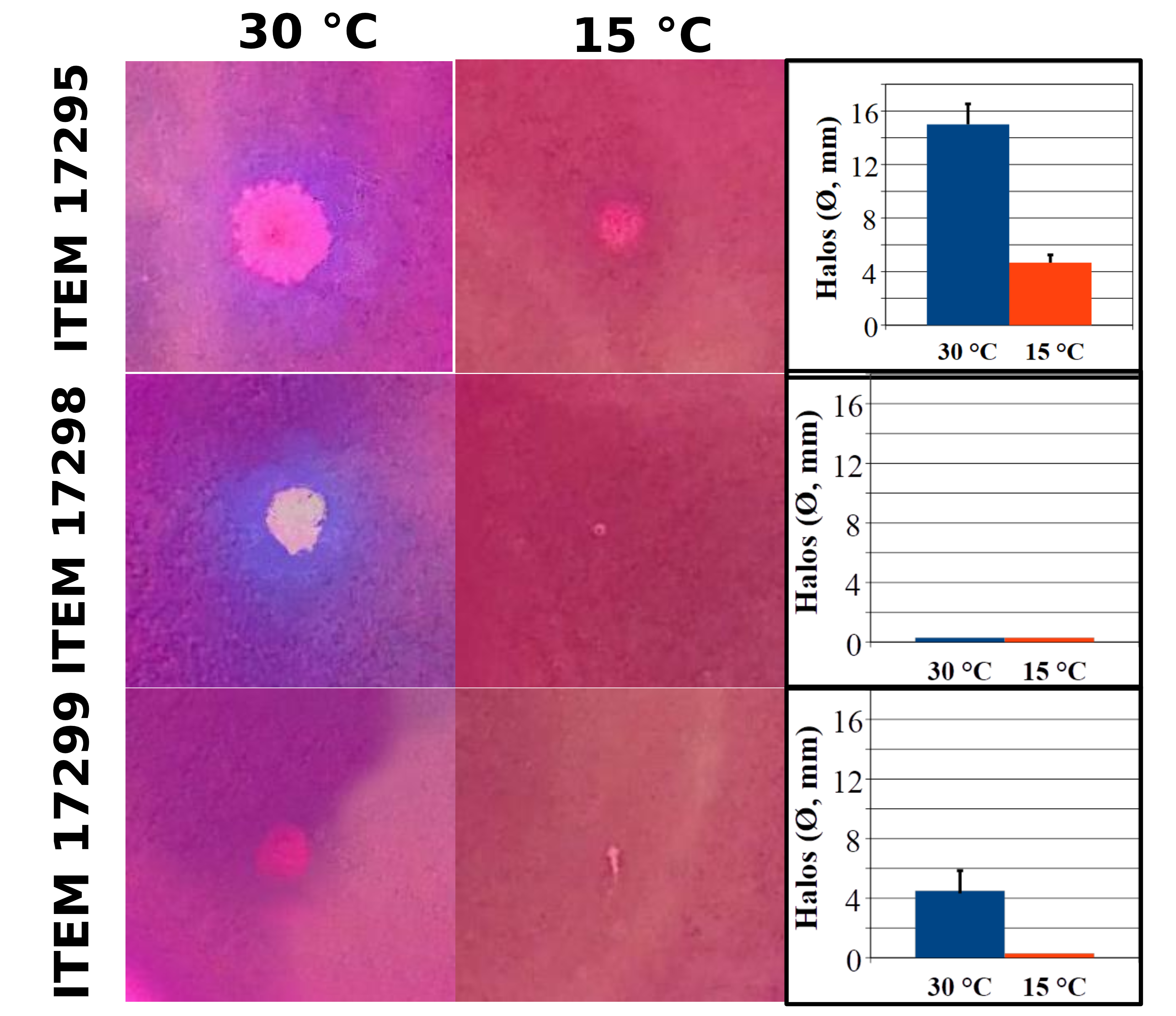

Supplement: Supplementary file 1 [file microorganisms-08-01208-s001.zip › Supplementary materials/Figure S5.png]
