# Supplementary material for: Genomic Analysis of Three Cheese-Borne Pseudomonas lactis with Biofilm and Spoilage-Associated Behavior
Source: Microorganisms. 2020 Aug 8;8(8):1208. doi: 10.3390/microorganisms8081208 (PMC7464908; doi:10.3390/microorganisms8081208)
Supplement: Supplementary file 1 [file microorganisms-08-01208-s001.zip › Supplementary materials/Table S6.doc]

**Table S6.** Spoilage operon in *P. lactis* strains

| ***Gene*** | **Product** | **ITEM 17298** | **ITEM 17295** | **ITEM 17299** |
| --- | --- | --- | --- | --- |
| *aprX* | serine protease AprX | PROKKA_00834 | GIB65_00575 | GIB64_22780 |
| *inh* | Proteinase inhibitor precursor | PROKKA_00835 | GIB65_00580 | GIB64_22785 |
| *prsD* | Type I secretion system ATP-binding protein PrsD | PROKKA_00836 | GIB65_00585 | GIB64_22790 |
| *prsE* | Type I secretion system membrane fusion protein PrsE | PROKKA_00837 | GIB65_00590 | GIB64_22795 |
| *tolC* | TolC family outer membrane protein | PROKKA_00838 | GIB65_00595 | GIB64_22800 |
| *prtA* | serine protease A | PROKKA_00839 | GIB65_00600 | GIB64_22805 |
| *prtB* | serine protease B | PROKKA_00840 | GIB65_00605 | GIB64_22810 |
| *lipA* | lipase A | PROKKA_00841 | GIB65_00610 | GIB64_22815 |
| *lipB* | lipase B | PROKKA_00842 | GIB65_00615 | GIB64_22820 |
