# Supplementary material for: Genomic Analysis of Three Cheese-Borne Pseudomonas lactis with Biofilm and Spoilage-Associated Behavior
Source: Microorganisms. 2020 Aug 8;8(8):1208. doi: 10.3390/microorganisms8081208 (PMC7464908; doi:10.3390/microorganisms8081208)
Supplement: Supplementary file 1 [file microorganisms-08-01208-s001.zip › Supplementary materials/Table S8.doc]

| **Table S8.** Lipases identified in *P. lactis* strains | | | | | |
| --- | --- | --- | --- | --- | --- |
| **Protein** | **Gene** | **Biological process** | **ITEM 17298** | **ITEM 17295** | **ITEM 17299** |
| Cardiolipin synthase B | *cls*B | cardiolipin biosynthetic process | PROKKA_03527 | GIB65_05800 | GIB64_14095 |
| Dermonecrotic toxin | *tox*A | phospholipase activity | PROKKA_01006 | GIB65_15670 | GIB64_10950 |
|  |  |  | PROKKA_01008 | GIB65_15660 | GIB64_10940 |
|  |  |  | PROKKA_02914 | GIB65_26435 | GIB64_23645 |
|  |  |  | PROKKA_04922 | GIB65_04295 | GIB64_12900 |
| Esterase EstA | *est*A | lipid biosynthetic process; bacterial-type flagellum-dependent cell motility; single-species biofilm formation | PROKKA_04708 | GIB65_21400 | GIB64_01800 |
| Esterase TesA | *tes*A | lipolysis | PROKKA_05351 | GIB65_08815 | GIB64_03485 |
| Lipase |  | lipolysis | PROKKA_00841 | GIB65_00610 | GIB64_22815 |
|  |  |  | PROKKA_01397 | GIB65_01305 | GIB64_28120 |
|  |  |  | PROKKA_05131 | GIB65_09710 | GIB64_18685 |
| Lipase 3 | *lip*3 | lipolysis | PROKKA_01538 | GIB65_04155 | GIB64_12745 |
| Monoacylglycerol lipase |  | lipolysis | PROKKA_05659 | GIB65_02105 | GIB64_07865 |
| NTE family protein RssA | *rss*A | lipolysis | PROKKA_01792 | GIB65_08150 | GIB64_17115 |
|  |  |  | PROKKA_04522 | GIB65_16815 | GIB64_24880 |
| Phospholipase C 2 | plcB | pathogenesis | PROKKA_03792 | GIB65_16425 | GIB64_15440 |
| Phospholipase YtpA | ytpA | antibiotic biosynthetic process | PROKKA_00641 | GIB65_24010 | GIB64_21085 |
|  |  |  | PROKKA_03475 | GIB65_22720 | GIB64_08675 |
| Thermostable monoacylglycerol lipase |  | lipolysis | PROKKA_04060 | GIB65_14265 | GIB64_01320 |
| Uncharacterized protein Rv0906 |  |  | PROKKA_05703 | GIB65_01875 | GIB64_08075 |
